# Cytoskeletal disorganization underlies PABPN1-mediated myogenic disability

**DOI:** 10.1038/s41598-020-74676-8

**Published:** 2020-10-19

**Authors:** Cyriel Sebastiaan Olie, Erik van der Wal, Domagoj Cikes, Loes Maton, Jessica C. de Greef, I.-Hsuan Lin, Yi-Fan Chen, Elsayad Kareem, Josef M. Penninger, Benedikt M. Kessler, Vered Raz

**Affiliations:** 1 Human Genetics Department, Leiden University Medical Center, Leiden, The Netherlands; 2 IMBA-Institute of Molecular Biotechnology of the Austrian Academy of Sciences, Vienna, Austria; 3 VYM Genome Research Center, National Yang-Ming University, Taipei, Taiwan; 4 College of Medical Science and Technology, Taipei Medical University, Taipei, Taiwan; 5 Advanced Microscopy Facility, Vienna Biocenter Core Facilities, Vienna Biocenter (VBC), Vienna, Austria; 6 Target Discovery Institute, Nuffield, Department of Medicine, University of Oxford, Oxford, UK

**Keywords:** Cytoskeleton, Mechanisms of disease

## Abstract

Muscle wasting and atrophy are regulated by multiple molecular processes, including mRNA processing. Reduced levels of the polyadenylation binding protein nucleus 1 (PABPN1), a multifactorial regulator of mRNA processing, cause muscle atrophy. A proteomic study in muscles with reduced PABPN1 levels suggested dysregulation of sarcomeric and cytoskeletal proteins. Here we investigated the hypothesis that reduced PABPN1 levels lead to an aberrant organization of the cytoskeleton. MURC, a plasma membrane-associated protein, was found to be more abundant in muscles with reduced PABPN1 levels, and it was found to be expressed at regions showing regeneration. A polarized cytoskeletal organization is typical for muscle cells, but muscle cells with reduced PABPN1 levels (named as shPAB) were characterized by a disorganized cytoskeleton that lacked polarization. Moreover, cell mechanical features and myogenic differentiation were significantly reduced in shPAB cells. Importantly, restoring cytoskeletal stability, by actin overexpression, was beneficial for myogenesis, expression of sarcomeric proteins and proper localization of MURC in shPAB cell cultures and in shPAB muscle bundle. We suggest that poor cytoskeletal mechanical features are caused by altered expression levels of cytoskeletal proteins and contribute to muscle wasting and atrophy.

## Introduction

Muscle wasting, the hallmark of muscle degeneration, is characterized by reduced muscle strength and impaired regeneration capacity^1^. Muscle wasting is associated with changes in multiple cellular mechanisms that affect protein homeostasis, transcription, protein acetylation and various metabolic pathways^2–5^. The regulation of muscle wasting can be caused by multiple molecular pathways. Others and we showed that reduced levels of polyadenylation binding protein 1 (PABPN1) cause muscle wasting, including muscle atrophy, extracellular matrix thickening, myofiber typing transitions and central nucleation^6,7^. Yet, the cellular mechanisms underlying PABPN1-mediated muscle wasting are not fully understood.

PABPN1 is a multifactorial regulator of mRNA processing^8^ and in cells with reduced PABPN1 levels, mRNA expression profiles are massively altered^9–11^. In human, PABPN1 levels are reduced from midlife onwards, which was found to be significant only in skeletal muscles^12^. An expansion mutation in PABPN1 is the genetic cause for oculopharyngeal muscular dystrophy (OPMD), a late onset myopathy that is characterized by muscle wasting (reviewed in: Brais, 2009). In OPMD, reduction of functional PABPN1 levels is caused by both aggregation of the expanded PABPN1 and reduced PABPN1 mRNA levels^13^. Those conditions can be modeled by the expression of shRNA to PABPN1, leading to reduced PABPN1 levels (named shPab in mouse and shPAB in human)^5,6^.

To elucidate the cellular mechanisms that are affected by reduced PABPN1 levels, proteome studies were generated from mouse models with reduced PABPN1 expression levels^5,7,14^. We found that the expression of sarcomeric and cytoskeletal proteins were significantly affected in the shPab mouse model^5^. The cytoskeleton provides structure and strength to the cell, and facilitates muscle contraction together with the sarcomeric proteins^15^. Disruptions in cytoskeletal arrangement, often due to mutations in cytoskeletal proteins, can cause various muscular dystrophies^16^. Also, in aged muscles a prominent disruption of the actin filament organization has been reported^17^. The actin filaments are an essential part in the contractile force of muscles^18^. In muscle cell culture, both microtubules and actin filaments have been suggested to be essential for myogenic differentiation^19,20^. It has been proposed that cross-talk between the actin-cytoskeleton and microtubules facilitates proper organization of the cytoskeleton^21^. Yet, the cytoskeletal organization in models for muscle wasting are not fully elucidated. Here we investigated the cytoskeletal structural changes that are dependent on PABPN1 levels using 2D and 3D models, and we discuss how these affect muscle wasting.

## Materials and methods

All methods in human and mouse tissues were carried out in accordance with guidelines and regulations after approval by the Leiden university medical centre ethical licensing.

### Mouse muscle tissue handling

All mouse muscles that were used in this study have previously been described by C. Olie et al.^5^. In brief, muscles denoted as shPab or control are tibialis anterior muscles injected with AAV9 particles expressing shRNA to *Pabpn1* or scrambled shRNA, respectively, as detailed by M. Riaz et al.^6^. AAV9 shPab and scrambled particles were injected contralateral into the left or the right leg, allowing paired analysis. This experimental design overcomes natural variations between mice. Five weeks after AAV9 injection muscles were harvested and directly frozen in liquid nitrogen. From each muscle cryosections were collected for protein analysis (mass-spectrometry and western blot) as previously described^5^.

### Human muscle tissue

Human vastus lateralis muscles were collected as waste material during knee surgery after an anterior cruciate ligament injury after obtaining an informed consent^12^. Cryosections of 16 µm thick were made with a cryostat CM3050S (Leica Microsystems) and pasted on Super Frost Plus glass slides (Menzel-Gläser; Thermo Fisher Scientific).

### Muscle cell culture

Immortalized human muscle cell (7304.1) shPAB and control stable cell lines were generated as previously described^12^. In brief, PABPN1 knockdown (shPAB) was generated after lentivirus transduction with a shRNA to *PABPN1*. The control cell line was transduced with scrambled shRNA^12^*.* Myoblast cells were maintained in growth medium (F10 medium supplemented with 15% FCS, 1 ng/ml bFGF, 10 ng/ml EGF and 0.4 ug/ml Dexamethasone). Differentiation (cell fusion) was conducted in DMEM supplemented with 2% horse-serum for four days. Stable actin-eGFP overexpressing cells were generated with a lentivirus transduction in control or shPAB cell cultures. The actin-eGFP was subcloned into a lentivirus expression cassette. Actin-eGFP expression was driven by the CMV promoter. All cultures were mycoplasma-free. Microtubule destabilization or stabilization treatments were performed using nocodazole (250 nM, Cayman Chemical) or paclitaxel (25 nM, Cayman Chemical), respectively for 2 h.

### Muscle bundle cultures

Muscle bundles were generated as previously described by Bakooshli et al. with minor adjustments^22^. Before generation of muscle bundles, bone-shaped PDMS chambers were sterilized and pre-treated with 1% Pluronic F-127 (Sigma-Aldrich) for 1 h at room temperature. Confluent myoblast cultures were dissociated with TrypLE Express Enzyme (Gibco) reagent and 3 × 10^5 cells were used per muscle bundle. Cells were incubated on ice and combined with an ice-cold hydrogel mixture consisting of 4 mg/ml bovine fibrinogen (Sigma-Aldrich) and 20% Growth Factor Reduced Matrigel (Corning). Before loading the cell/hydrogel mixture into a bone-shaped PDMS chamber, 0.8 units of Bovine Thrombin (Sigma-Aldrich) were added to initiate fibrinogen polymerization. Loaded PDMS chambers were incubated for 30 min at 37 °C and after polymerization muscle bundles were kept in normal proliferation medium containing 1.5 mg/ml 6-aminocaproic acid (6-ACA) (Sigma-Aldrich). After 2 days, differentiation was induced by changing medium to differentiation medium (DMEM high glucose (Gibco), 2% horse serum (Gibco), 1% Penicillin G (Sigma-Aldrich) and 2 mg/ml 6-Aca). Seven days after differentiation muscle bundles were fixed overnight at 4 °C with 2% PFA. Muscle bundles were stored in PBS at 4 °C.

### Protein analysis

Proteins from tibialis anterior muscles were extracted as previously described^5^. Proteins from human muscle cell culture were extracted using RIPA extraction buffer (20 mM Tris, pH 7.4, 150 mM NaCl, 5 mM EDTA, 1% NP40, 5% glycerol and 1 mM DTT and protease inhibitor cocktail). The extracts were briefly sonicated and protein concentrations were determined using Bio-Rad protein assay. Subcellular fractionation was done as followed: cells were trypsinized and were homogenized with a douncer at 4 °C in lysis buffer (150 mM NaCl, 50 mM HEPES pH 7.4, 50 ug/mL Digitonin, 1 M Hexylene glycol and protease inhibitor cocktail). Centrifugation at 4,000 × *g* separated the organelles and nuclei from the cytosolic fraction. Ultracentrifugation at 160,000 × *g* for 90 min then separated the heavy and light membranes (including the plasma membrane) from the cytosolic proteins. Protein extracts were separated using pre-casted SDS-PAGE (Criterion XT, Bio-Rad). Western blot was carried out using PVDF membranes and blotted with the following antibodies: actin (sc-8432; Santa Cruz), CSRP3 (GTX110536, GeneTex), GAPDH (MA5-15,738, Thermo Fisher Scientific), MURC (HPA021021, Atlas Antibodies), PABPN1 (LS-B8482, LSBio, USA) and Tubulin (T6199; Sigma-Aldrich). Primary antibodies were labeled using fluorescent IRDye 800CW or IRDye 680RD (LI-COR) and were subsequently detected using the Odyssey CLx Infrared imaging system (LI-COR, NE. USA). Protein expression levels were quantified using ImageJ (https://imagej.nih.gov/ij/). All expression levels were corrected for background and normalized to loading controls. All images of blots that are presented in the same panel are from the same experiments. Quantification was made from multiple experiments.

### Cell mechanical properties

Probing of cell mechanical properties were carried out with Brillouin light scattering microscopy and atomic force microscopy. A day before analysis, cells were seeded on Matrigel Coated 35 mm glass bottom microwell dishes (MatTek Corporation) in growth media at a density of 60–50%. During imaging, samples were kept at 37 °C and 5% CO_2_ levels via an onstage heater.

The Brillouin light scattering microscopy was performed using a home built Brillouin confocal microscope described in^23^. Excitation was via a single mode 532 nm laser (Torus, Laser Quantum, DE). For all scans, the laser power at the sample was between 1–3 mW, and the swell time per point, which was also the acquisition time of each spectra, was 100 ms. The spectral projection was measured on a cooled EM CCD camera (ImageEMX II, Hamamatsu, JP). The spectrometer was coupled to an inverted microscope frame (IX73, Olympus, JP) via a physical pinhole to assure confocal detection. Spectral phasor analysis^24^ was used to obtain initial parameter estimates for peak positions and widths which were subsequently inserted into a non-linear least squares fitting algorithm that fitted two broadened Lorentzian functions (Voigt functions) to obtain the two peak positions, from which the Brillouin Frequency Shift could be calculated. The Brillouin Frequency Shift is directly proportional to the (longitudinal) acoustic phonon speed, which has been empirically found to be higher for "stiffer" samples and smaller for "softer" samples. All data analysis was performed in Matlab (MathWorks, DE) using custom written scripts^23^. The Atomic force microscopy was performed on a JPK nanowizard 4 system. Applied imaging mode used was QI (quantitative imaging) where a force curve is applied at each point. Area analyzed per each cell was 5 µm × 5 µm (64 × 64 pixels) with an approach speed of 35 µm/s (3.4 ms/pixel), and applied forces of up to 118 pN. The cantilevers used for sample probing (qp-BioAC, CB3, Nanosensors) were calibrated to 828 µN/m as a force constant. All data analysis was performed in JPK SPM data processing software.

### Immunofluorescence

Immunostaining on muscle sections was carried out with the previously described protocol^5^ using the following primary antibodies; CSRP3 (GTX110536, GeneTex), Dystrophin (ab7164, Abcam), MURC (HPA021021, Atlas Antibodies) and specific myosin heavy chain isotype were stained with MyHC-2A and MyHC-2B conjugated to 594 and 488 fluorophore, respectively^25^. Secondary antibodies used were anti-rabbit conjugated to Cy5 or anti-mouse conjugated with Alexa 488 (Thermo Fisher Scientific). Immunofluorescence in cell cultures was carried out after fixation in 2% formaldehyde and permeabilization in 1% triton-X100. Primary antibodies used for immunofluorescent staining in cell culture were actin (sc-8432; Santa Cruz), MURC (HPA021021, Atlas Antibodies) and tubulin (T6199; Sigma-Aldrich). Fused cells (myotubes) were visualized using an antibody against Myosin heavy chain isoforms (MyHC) (MF20, Sigma-Aldrich). Primary antibodies were visualized with anti-rabbit or anti-mouse conjugated with Alexa 488 or Cy5 (Thermo Fisher Scientific). DAPI was used to stain nuclei in both muscle tissue and cell culture.

Immunofluorescence of muscle bundles was carried out with the following protocol: After washing with PBS muscle bundles were incubated in a blocking buffer (3% BSA, 0.3% Triton X-100, 0.1% Tween-20 in PBS) for 1 h and washed in PBS. An incubation with the primary antibody was carried out for one hour in antibody buffer (0.1% BSA, 0.3% Triton X-100, 0.1% Tween-20 in PBS). After PBT washing, secondary antibody mix containing DAPI was incubated for 30 min in antibody buffer. After PBT washing muscle bundles were imaged with the Dragonfly 2-photon imaging system; ANDOR Oxford Instruments.

### Imaging and image quantification

Imaging of the mouse and human tissue sections was carried out with the Leica DM5500B fluorescent microscope with a Leica Kubler as a light source equipped with a black and white camera DFC360FX v. FCAM2, and the following Leica filter cubes: A4, L5, TxR and Y5. Images were taken with 10x, 20 × or 63 × objectives using single pass filter cubes and bright field images using the differential interference contrast, at a display lookout table (LUT) covering the full range of the data. Images were captured with the Leica application suite X version: 3.3.3.16958. Image quantification from a muscle tissue was made with ImageJ. The mean fluorescence intensity within myofibers was measured from each channel separately from over 400 myofibers covering the entire tissue. Merged images were made with the Leica application suite and adjustments of individual colour channels was made for visualization only. Images were acquired at a resolution 1024 × 1024 and the resolution was not changed during downstream processing.

Imaging of cells and muscle bundles was carried out using the Arrayscan VTI HCA, Cellomics, equipped with a LED as a light source with a black and white camera (Thermo Fisher Scientific). Cells were imaged on a 96 well plate plates using a 10X, 20X or 40 × objectives. Cell-based image quantification analyses were carried out with HCS Studio Cell Analysis Software V (Thermo Fisher Scientific). The colocalization toolbox was used to calculate fusion index, which is the fraction of segmented nuclei in segmented MyHC objects versus the total number of nuclei. The spot detection toolbox was used to segment MURC foci using a threshold focusing only on the MURC foci. A uniform image processing and image quantification setting was used to compare control and shPAB cells. Merged images were made for visualization only using pseudo colouring.

### Statistical analysis

A correlation between myofiber mean fluorescence intensity was calculated using Pearson correlation. Significance was assessed using parametric t-tests. The data used from the shPab proteome was assessed for significance using a paired ratio t-test. The paired t-test overcomes any variation between mice as previously described^5^. The in vitro experiments were tested using a student’s t-test.

## Results

### Reduced PABPN1 expression levels highly affect structural proteins

**Figure 1:**
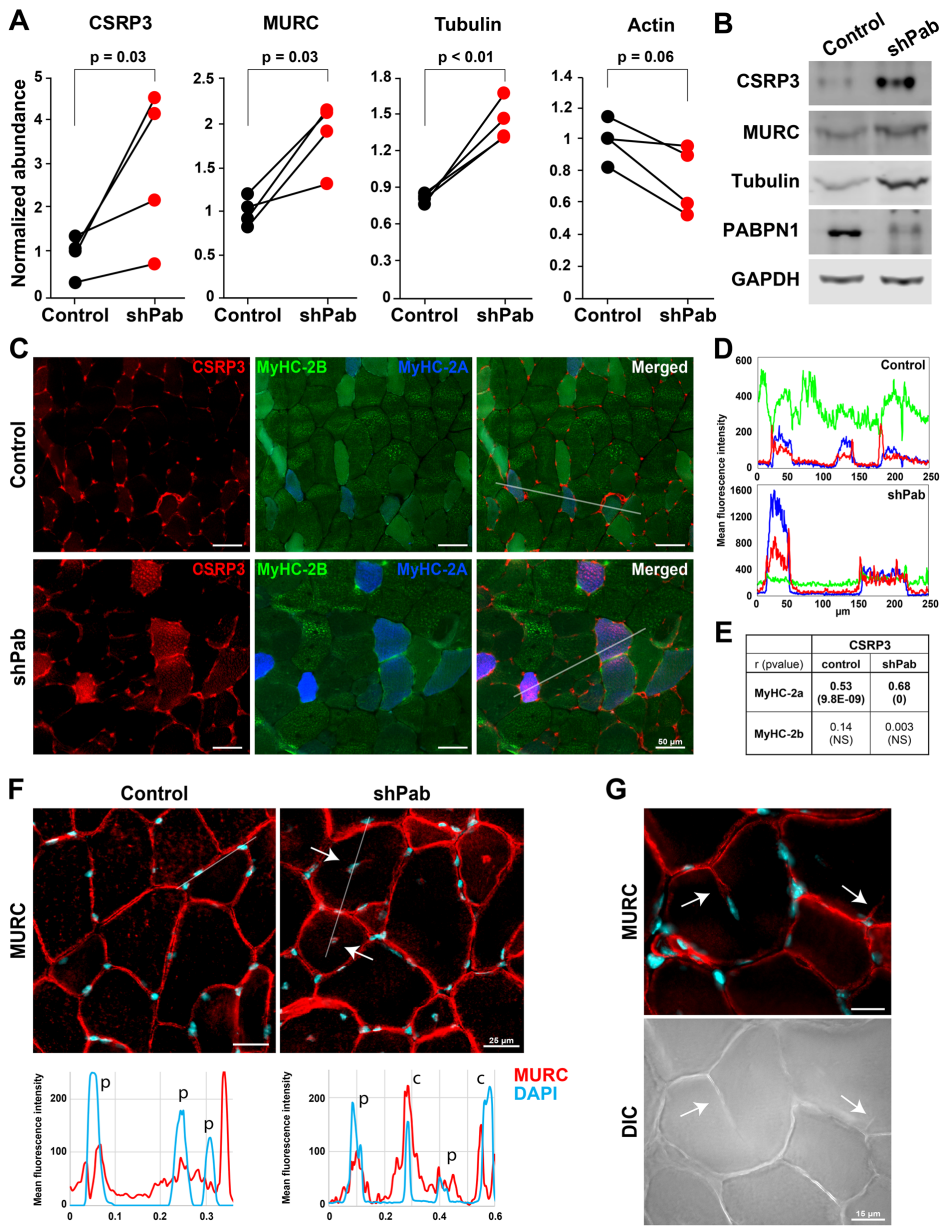
Expression levels and spatial expression of CSRP3 and MURC in shPab muscles. **A** Paired dot-plots show the normalized (median subtracted) mass spectrometry abundances of CSRP3, MURC, tubulin, and actin in control and shPab muscles. A statistical difference was assessed with a paired ratio t-test. (N = 4 mice). **B** Western blots show expression levels in paired muscles. GAPDH was used as loading control. PABPN1 level confirms down-regulation. Full-length blots are presented in Fig. S10. **C** Spatial localization of CSRP3 in control and shPab muscles. Images from a representative region in tibialis anterior muscle, show MyHC-2B and MyHC-2A transitions between control and shPab. Staining shows CSRP3 in red, MyHC isoforms type-2A in blue and type-2B in green. The merged image shows CSRP3 enrichment in type-2A myofibers. Scale bar is 50 µm. Transparent line indicates area used for distribution plots. **D** Distributions plot shows the mean fluorescence intensity of CSRP3 (in red), MyHC-2A (blue) and MyHC-2B (green). Upper panel corresponds to the transparent line found in the control merged image and lower panel for the transparent line found in shPab merged image. **E** Table indicates a Pearson correlation assessment of CSRP3 mean fluorescence intensity with myosin heavy chain MFI. MFI of CSRP3 and MyHC-2A and -2B was measured from all myofibers in cross-sections of control (210myofibers) and shPab (300myofibers) muscles. **F** Spatial localization of MURC in shPab muscles. Upper panel shows representative images in tibialis anterior: MURC is depicted in red and DAPI nuclear counterstaining in cyan. MURC staining in central nuclei is shown in the fluorescence distribution plot. Arrows point to central nuclei. Scale bar is 25 µm. Lower panel shows intensity distribution of MURC and DAPI along the white line (in the upper panel) peripheral nuclei and depicted with *P*, and central nuclei with *C*. **G** MURC signal in a myofiber splitting initiation (left, depicted by arrows) in shPab muscle. The same regions are indicated by arrows in a differential interference contrast (DIC) image (right). Scale bar is 15 µm.

In our previous proteome study we found that the cytoskeleton gene ontology term was significantly enriched in shPab muscles^5^. In total, the expression of 34 cytoskeletal proteins were significantly altered in shPab muscles compared to control muscles (Table S1). To elucidate the effect of PABPN1 on the cytoskeleton organization in muscle cells, we selected cytoskeletal proteins that showed a fold change of 1.5 or higher, or lower than 0.67 (Table S1). Subsequently, we focused on candidates whose fold change highly correlated with PABPN1 down-regulation levels (Table S1). Amongst those proteins, 43% were categorized to the actin cytoskeleton functional group and 25% to microtubule functional group (Table S1). We noticed that tubulin expression levels were found to be significantly elevated, whereas actin showed a reduced expression in shPab muscles (Fig. 1A). This finding is in agreement with a recent transcriptome study of skeletal muscles in aged mice reported that the cytoskeleton gene ontology group was also significantly affected^26^. We then, compared the significantly affected cytoskeletal proteins in the shPab proteome with the aged muscles transcriptome and found that in addition to tubulin- and actin-associated proteins, the fold change of the Cysteine and glycine-rich protein 3 (CSRP3) and the Muscle-related coiled-coil protein (MURC also known as Caveolae-associated protein 4 (Cavin4)) were increased in both studies (Table S1). Moreover, fold changes of these proteins highly correlated with PABPN1 fold changes (CSRP3 r = -0.89; MURC r = -0.98). A western blot confirmed the changes in expression in shPab muscles (Fig. 1B and Fig. S1). We selected CSRP3 and MURC for further analysis. CSRP3 is an actin binding protein that was shown to be involved in myogenesis^27,28^. A mutation in CSRP3 was found as the cause of hypertrophic cardiomyopathy^29^. MURC was also reported to be required for myogenic differentiation^28,30^.

To assess if the expression of both genes is directly regulated by PABPN1, we investigated the alternative polyadenylation site (APA) usage, which is a direct functional readout for PABPN1 function^9^. We previously published the genes with a PABPN1-mediated APA in tibialis anterior muscles^10,11^. Here we show that *Murc* APA at the 3′-UTR is under PABPN1 regulation (Fig. S2). In contrast, in *Csrp3* only one polyadenylation site was found over the entire gene (Fig. S2), suggesting that *Csrp3* levels are not directly regulated by PABPN1. The expression levels of CSRP3 and MURC showed high variation between mice (Fig. 1A). Since the expression of both proteins highly correlated with PABPN1 levels, this variation could be due to variations in PABPN1 levels between muscles.

Next, we investigated the localization of MURC and CSRP3 in the shPab muscles using immunohistology. CSRP3 in shPab muscle cross-sections was found in distinguished myofibers (Fig. 1C). Since MyHC-2A expression in myofibers is increased in shPab^6^, we assessed a colocalization of CSRP3 with MyHC-2A and MyHC-2B. We found that CSRP3 was highly expressed in MyHC-2A positive myofibers, including myofibers that expressed both MyHC-2A and MyHC-2B isoforms (Fig. 1C-D and Fig. S3). A correlation assessment of the mean fluorescence intensity in individual myofibers showed that the mean fluorescence intensity of CSRP3 correlated with that of MyHC-2A but not with MyHC-2B (Fig. 1E). Consistent with a higher proportions of MyHC-2A positive myofibers in shPab muscles^6^, the correlation between MyHC-2A and CSRP3 was higher in shPab compared to control muscles (Fig. 1E, r = 0.68; r = 0.53, respectively). In contrast, MURC spatial colocalization with MyHC-isoforms was not observed (Fig. S4), and therefore we did not assess a correlation between MURC and MyHC-isoforms in myofibers.

**Figure 2:**
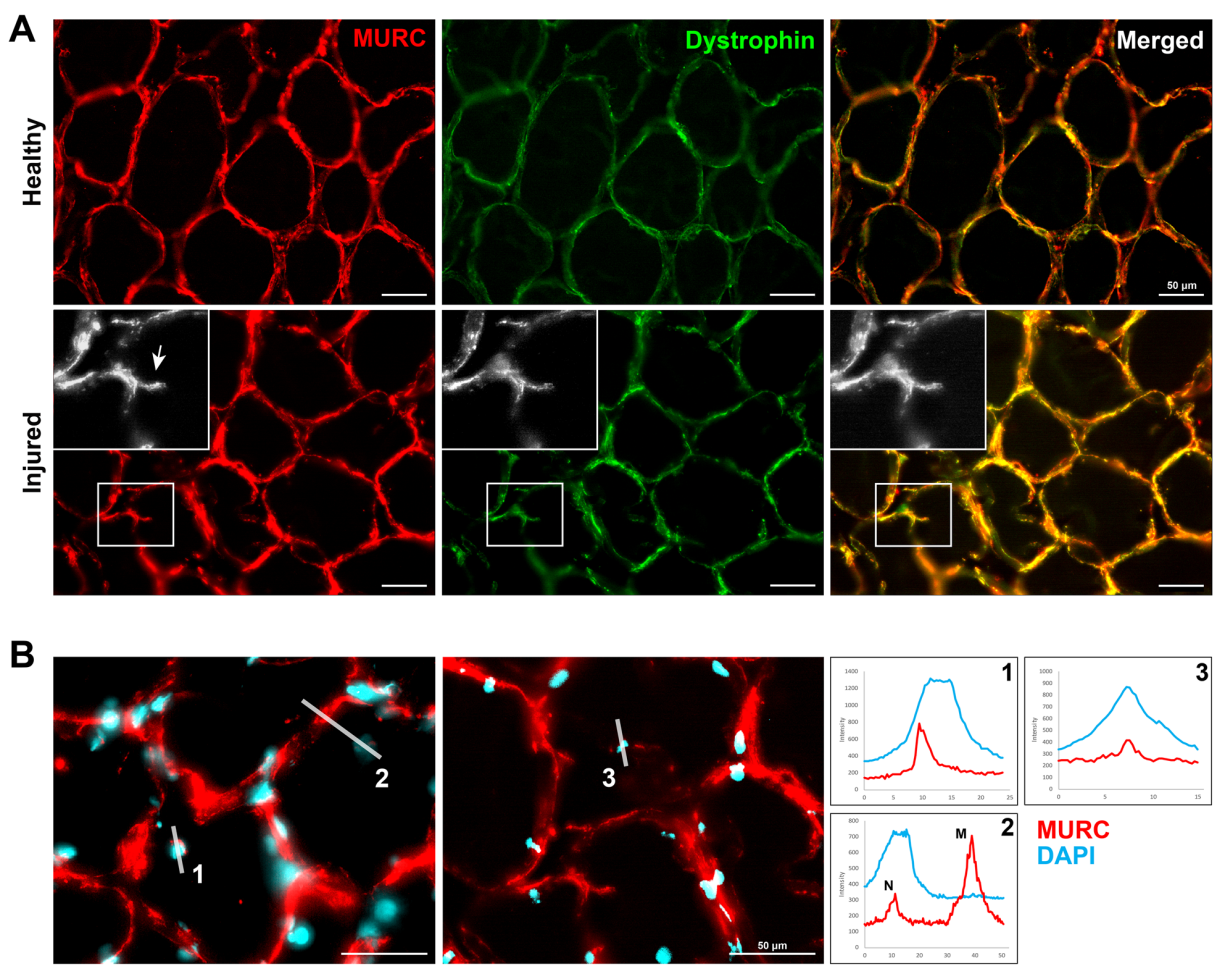
Cytoskeletal changes in human skeletal muscles are also characterized by MURC expression. **A** Immunohistochemistry of human muscle cross-sections. Images represent healthy or injured regions. MURC is stained in red and dystrophin is stained in green, an overlap is in the merged image. A myofiber splitting, pointed by an arrow, is shown in the enlarged grayscale image, and the corresponding region is highlighted with a square. Scale bar is 50 µm. **B** MURC staining in central nuclei in wounded muscles is shown in two muscles. MURC is depicted in red, and nuclei with cyan. For three regions (marked 1–3 with a white line), a corresponding intensity distribution plot is shown in the right panel. MURC is in red, and DAPI in cyan. Scale bar is 50 µm.

MURC signal was localized to the periphery of the myofibers in both control and shPab muscles (Fig. 1F), which is in agreement with its membrane localization^31^. In shPab muscles MURC localization was also found to colocalize with central nuclei (Fig. 1F). An increase in the number of central nuclei is one of the histological features of shPab muscles^5,6^. CSRP3 expression was not found in central nuclei (Fig. S5). Interestingly, MURC staining was also found to co-localize with newly formed membranes (also known as myofiber splitting or branching^32^) in between myofibers, which were predominantly found in shPab muscles (Fig. 1G). Regions of splitting myofibers are also enriched in injured muscles^32^. To verify MURC spatial expression in regions of splitting myofibers, we stained human vastus lateralis muscles that were collected from young athletes during a knee surgery. We found MURC expression in central nuclei and in splitting myofibers in injured regions but not in intact regions (Fig. 2). Confirming previous studies showing MURC plasma membrane localization^31,33^, we show MURC colocalization with dystrophin in human vastus lateralis muscles (Fig. 2). This could suggest that MURC expression is associated with muscle regeneration.

### MURC localization marks an aberrant cell fusion in shPAB cultures

**Figure 3:**
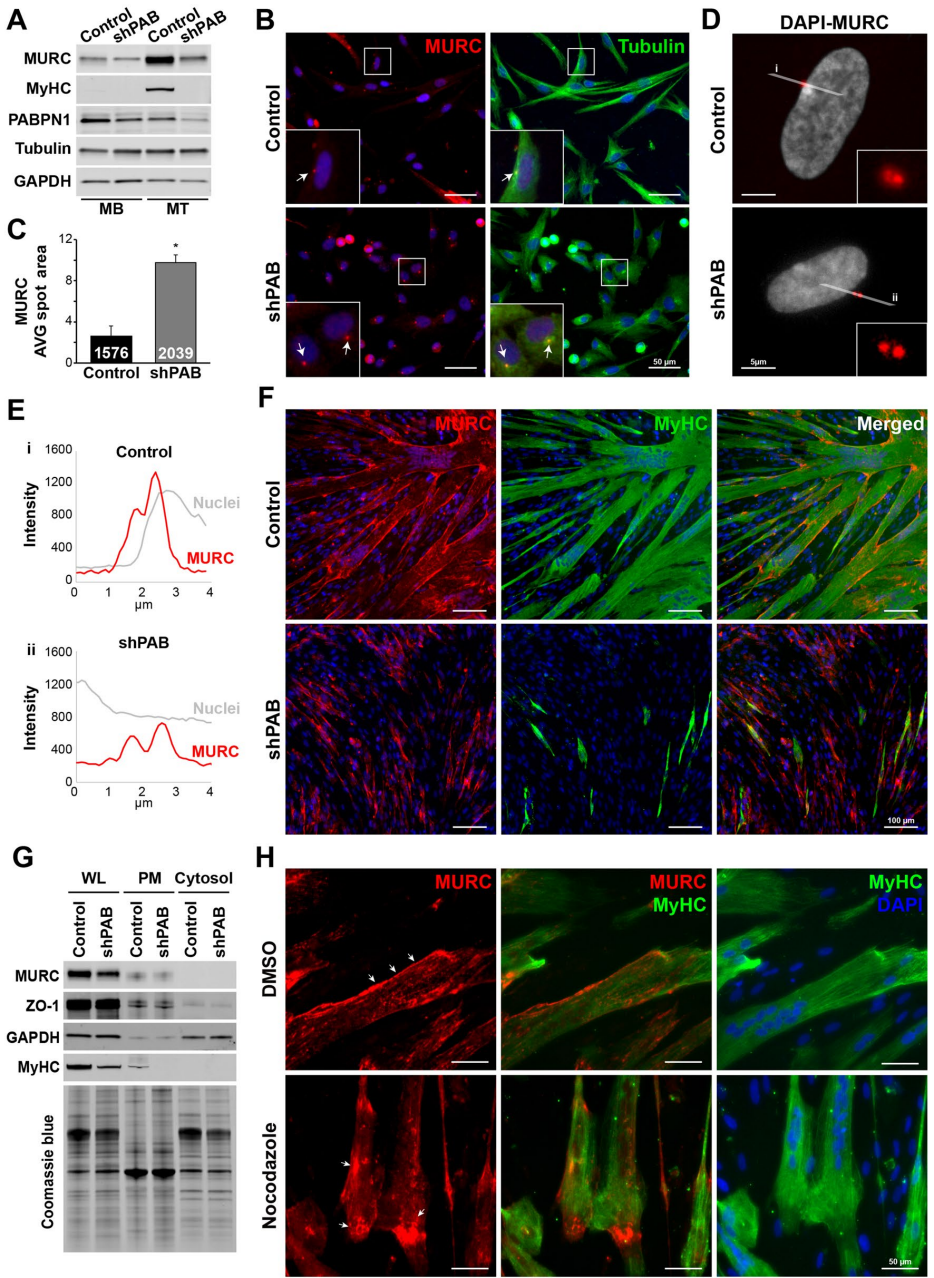
MURC localization in shPAB human muscle cell cultures. **A** Western blot shows MURC expression levels in myoblast and myotubes, respectively. GAPDH was used as loading control. PABPN1 level confirms down-regulation. Full-length blots are presented in Fig. S10. **B** Representative images of control and shPAB human muscle cell cultures. Control and shPAB myoblasts are stained with tubulin (green) and MURC (red). White arrows point to MURC foci and the colocalization with tubulin. Nuclei are counterstained in blue with DAPI. Scale bar is 50 µm. **C** Bar chart shows average MURC foci area in control and shPAB cultures. Numbers inside bars depict the number of nuclei, averages and standard deviations are from (N = 3). Statistical significance was assessed with the student’s t-test **D** Images show MURC foci in red and nuclei are counterstained with DAPI in white. Grey lines (i or ii) indicate the region of which the mean fluorescence intensity overlay is shown in **E**. **F** Control and shPAB fused cultures are stained for MyHC (green) and MURC (red). Merged images show overlay between MyHC and MURC. A nuclear counterstaining is in blue. Scale bar is 100 µm. **G** Western blot shows input (total lysate; WL), plasma membrane (PM) and cytosolic fractions in differentiated control and shPAB cell cultures. ZO-1 was used as a plasma membrane (PM) marker and GAPDH was as a cytosolic marker. Coomassie blue staining shows loading control. The full-length blots are presented in Fig. S10. **H** Images show MURC (in red) localization in mock-treated and nocodazole-treated myotubes (depicted with MyHC in green). White arrows indicate MURC localization at the plasma membrane. Scale bar is 50 µm.

To elucidate a role for MURC in PABPN1-mediated myogenic defects, we studied MURC expression in a stable shPAB human muscle cell culture. MURC expression levels in human myoblast culture were low compared to myotube cell culture (Fig. 3A), which is in agreement with previous studies^30,31^. MURC immunofluorescence staining in myoblasts showed a predominant localization in foci, one per cell (Fig. 3B). MURC foci were found to colocalize with tubulin foci (Fig. 3B). Using a high-throughput image quantification we found that the average area of the MURC foci in shPAB myoblasts was significantly larger compared with control (Fig. 3C). Higher resolution imaging showed that MURC foci were in fact two adjacent foci, yet the distance between the two foci was larger in shPAB myoblasts (Fig. 3D-E). In mitotic cells, tubulin foci were reported to be associated with centrosomes, playing a role in microtubule organization and spindle formation in mitotic cells^34,35^. In agreement, in dividing cells we found MURC localization to the spindles (Fig. S6). Together this suggests that MURC might also associate with the centriole.

In fused cultures MURC levels were elevated compared to myoblast cultures (Fig. 3A), and immunofluorescence showed that MURC outlined myotubes in control cell culture (Fig. 3F). Myotubes were recognized by the expression of MyHC, and MURC outlines the myotubes in control cells. (Fig. 3F). Subcellular fractionation confirmed MURC to be enriched at the membrane fraction compared with the cytosolic faction (Fig. 3G). In shPAB cell culture that was incubated with fusion media MURC expression did not overlap with MyHC (Fig. 3F and Fig. S7). Multinucleated myotubes expressing MyHC were hardly detected in shPAB cultures (Fig. 3F). Assessment of the myogenic differentiation kinetics further demonstrated that myotube formation and maturation was concomitant with increased MURC expression in control but not in shPAB cell culture (Fig. S7). Although MURC in shPAB culture was detected at the membrane fraction (Fig. 3G), immunofluorescence showed that MURC signal did not outline the cell (Fig. 3F). This suggests that a proper MURC localization to the myotube plasma membrane is abolished in cultures with reduced PABPN1 levels. Trafficking and protein association to the plasma membrane can be stabilized by the microtubules^36^. Therefore, we investigated if MURC plasma membrane localization is affected by microtubule stability. Treatment with nocodazole, a β-tubulin binding compound that depolarizes microtubules, disrupted MURC localization at the plasma membrane in fused cell cultures (Fig. 3H). This suggests that MURC dis-trafficking to the plasma membrane in shPAB is associated with aberrant microtubule organization.

### Reduced PABPN1 levels in muscle cells lead to an aberrant cytoskeletal architecture and reduced cell tension

**Figure 4:**
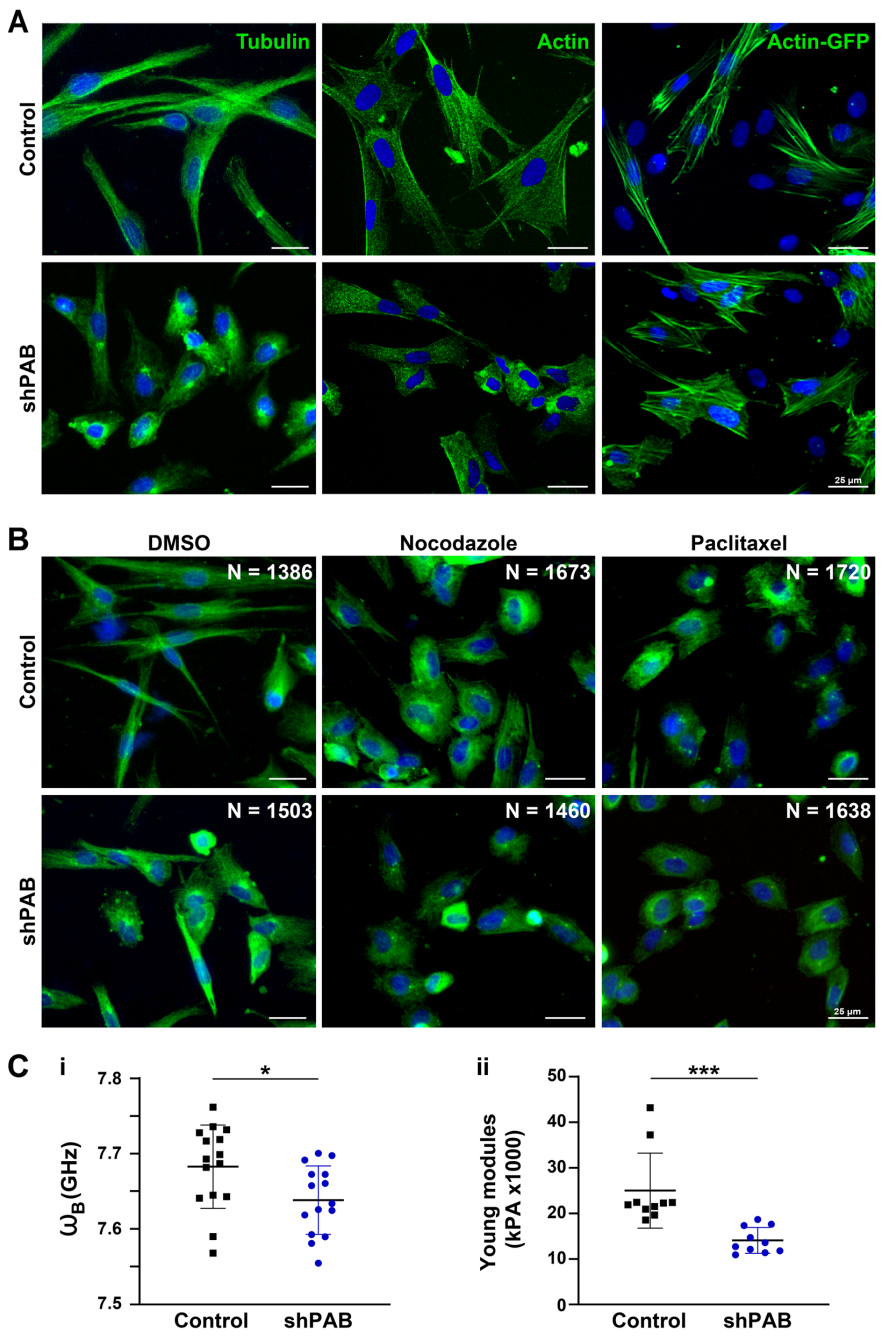
Disrupted cytoskeletal spatial organization in shPAB human muscle cell cultures. **A** Representative images of control and shPAB human muscle cell cultures, stained with antibodies to tubulin and actin, and the actin filaments were visualized with actin-GFP. **B** Tubulin staining in control and shPAB myoblast cell cultures after DMSO, 100 nM nocodazole or 25 nM paclitaxel treatment for 2 h. Scale bar is 25 µm. **C** Measurements of cell-mechanics properties in control and shPAB cells using the Brillouin Light Scattering Microscopy (Ci) or the Atomic Force Microscopy (Cii). Measurements were carried out in myoblasts; every dot represents the median from 1000 measurements in a cell. Cell stiffness is measured by GHz, and the young modulus reports the cell surface tension. Averages and standard deviations are from N = 15 cells. Statistical significance was calculated with the student’s t-test.

Cells with reduced PABPN1 levels are characterized by reduced myogenesis^12,37^, but the cellular causes have not been investigated. As dynamic cytoskeletal reorganization plays a role in myogenesis^38^, we investigated the cytoskeletal organization in shPAB cell culture and whether myogenesis could be enhanced by modulation of the cytoskeleton. In control myoblasts both tubulin and actin immunofluorescence staining showed elongated filaments in the longitudinal cell axis (Fig. 4A). The shPAB myoblasts lacked those structured filaments and the cells were more rounded in comparison to control myoblasts (Fig. 4A). We then visualized actin filaments with an overexpression of actin-GFP. Whereas in control cells, the actin filaments were stretched and parallel organized along the longitudinal axis, in shPAB cells the actin filaments were disorganized and unstructured (Fig. 4A). A rounded cell shape was also observed after treatment of myoblasts with drugs that modulate microtubule stability (Fig. 4B). Together, this suggests that a polarized cytoskeletal organization was not formed or maintained in shPAB myoblasts, which directly influences biophysical cell properties. We then measured the mechanical cell properties in both control and shPAB cell cultures using two different methods. The Brillouin light scattering microscopy measures the cell its hydrostatic pressure and cytoplasmic viscoelasticity, which was found to be lower in shPAB cells (Fig. 4Ci). The atomic force microscopy measures cell surface tension, which was also found to be significantly lower in shPAB cells (Fig. 4Cii). With the atomic force microscopy larger differences and smaller variations were found because the measurements are more focal compared with the Brillouin light scattering microscopy. Reduced stiffness, hydrostatic pressure and cytoplasmic viscoelasticity are consistent with the observed reduced cytoskeletal filament organization in shPAB cells.

**Figure 5:**
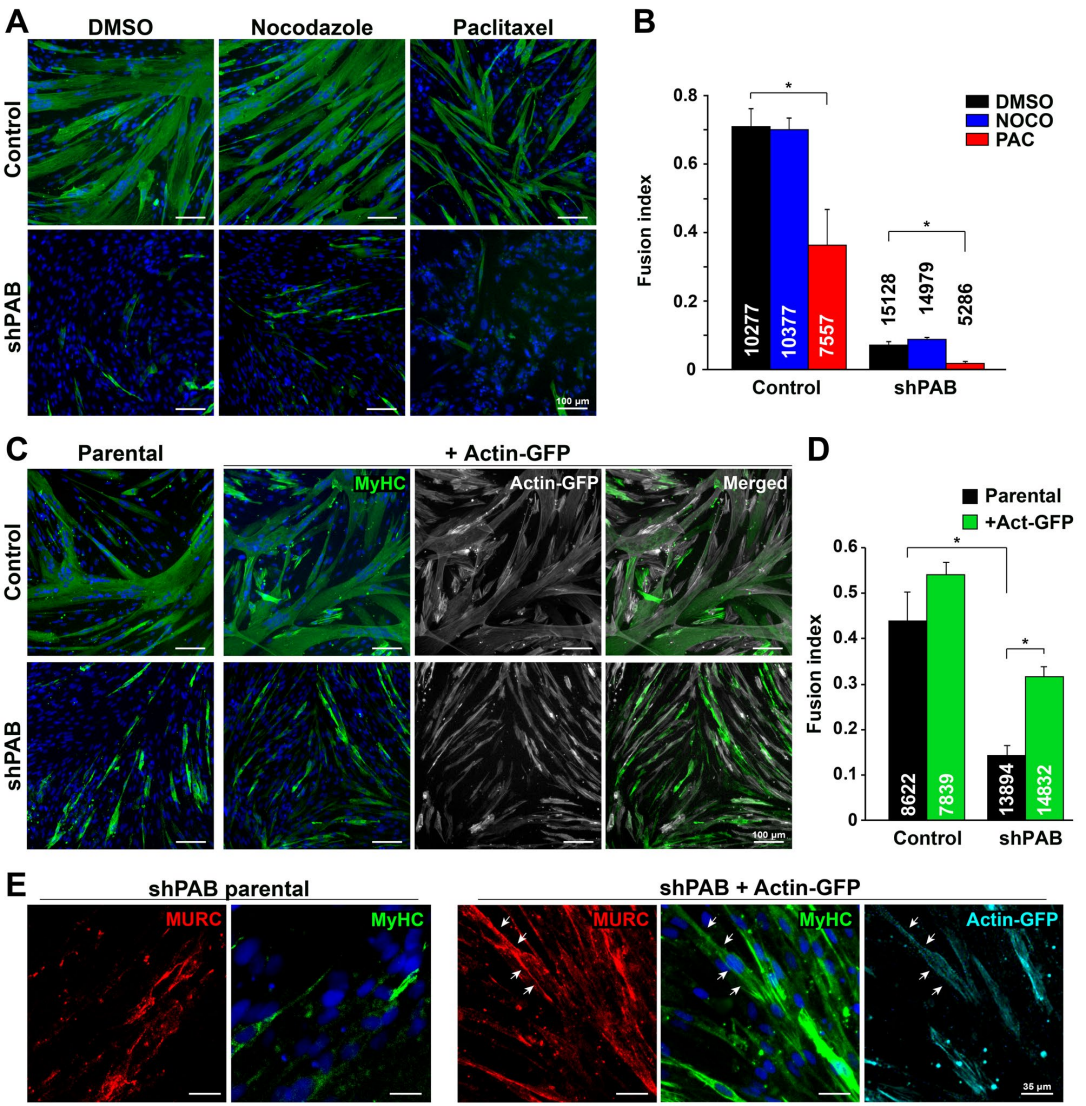
Reduced myogenesis in shPAB is affected by cytoskeletal stabilization and organization. **A** Representative images of fused control and shPAB cultures. MyHC staining is depicted in green and the nuclei in blue. **B** Bar chart shows fusion index, which was calculated by the fraction of nuclei within MyHC objects. The average number of nuclei per replicate is denoted in or above each bar. Average and standard deviation are from four replicates. Scale bar is 100 µm. **C** Cell fusion in the actin-GFP expressing cell lines. Representative images of cell cultures fused for four days, parental or actin-GFP overexpressed control or shPAB cell lines. MyHC is depicted in green and actin-GFP in gray, the merged image shows an overlay between MyHC and actin-GFP. **D** Bar chart shows fusion index in parental and actin-GFP control and shPAB cultures. Average and standard deviation are from three replicates. The average number of nuclei per replicate is denoted in each bar. Scale bar is 100 µm. **E** Images show MURC localization (depicted in red) in the actin-GFP (depicted in cyan) fused cell cultures. Scale bars is 35 µm. Statistical significance was assessed using a student’s T-test, * denotes p < 0.05. Every experimental setup was repeated three times.

We then investigated if reduced cytoskeletal organization affects cell fusion in human muscle cell culture. To test this, myoblasts were treated with drugs affecting microtubule stability and were subsequently incubated with fusion medium for four days. In contrast to the destabilizing effect of nocodazole on microtubules, paclitaxel stabilizes cytoskeletal filaments by binding to αβ-tubulin heterodimers. Paclitaxel treatment led to a reduction in cell number and consequently to a lower fusion index in both control and shPAB cell cultures (Fig. 5A-B). Moreover, after paclitaxel treatment the MyHC-positive cells were narrower and contained fewer nuclei compared to untreated control myotubes (Fig. 5A). As myotube formation depends on cell density, we then treated matured myotubes with paclitaxel for two hours. After this paclitaxel treatment myotubes also displayed narrow myotubes containing fewer nuclei (Fig. S8). In contrast, a two-hour treatment with nocodazole, a microtubule destabilizing agent, had no obvious effect on fused cells or on the fusion index (Fig. 5A-B). As the effect of nocodazole is reversible^39,40^, this suggests that microtubule destabilization does not play a role in the first phase of cell fusion. In line with nocodazole treatment of myoblasts before fusion, nocodazole treatment on fully matured myotubes also had little affect (Fig. S8). Together, this data suggests that during the first phase of cell fusion, destabilization of microtubule filaments but not stabilization, might play a role.

To further investigate the hypothesis that cytoskeletal organization affects myogenesis in shPAB cells, we studied the ability to fuse in control or shPAB cell lines that stably expressed actin fused to GFP. The percentage of GFP-positive cells was similar between control and shPAB cultures (Fig. S9), ensuring that any differences in cell fusion were not due to differences in lentivirus transduction efficiency. In control cells, actin-GFP expression did not affect fusion index (Fig. 5C-D). In contrast, MyHC expression in shPAB cell cultures significantly increased by actin-GFP expression (Fig. 5C-D). However, those MyHC-expressing cells were not multinucleated and were narrower compared to control myotubes (Fig. 5C). We then further characterized the myotubes in the shPAB + actin-GFP expressing cell culture by studying MURC expression. MURC signal was found to co-localize with the membranes of MyHC-expressing cells in shPAB + actin-GFP cell culture (Fig. 5E). This colocalization to membranes in fused cultures was absent in the parental shPAB cultures (Fig. 5E). Together, this data suggests that stabilizing the actin filaments could enhance myogenesis and restore MURC localization at the myotube plasma membranes in shPAB cell culture.

**Figure 6:**
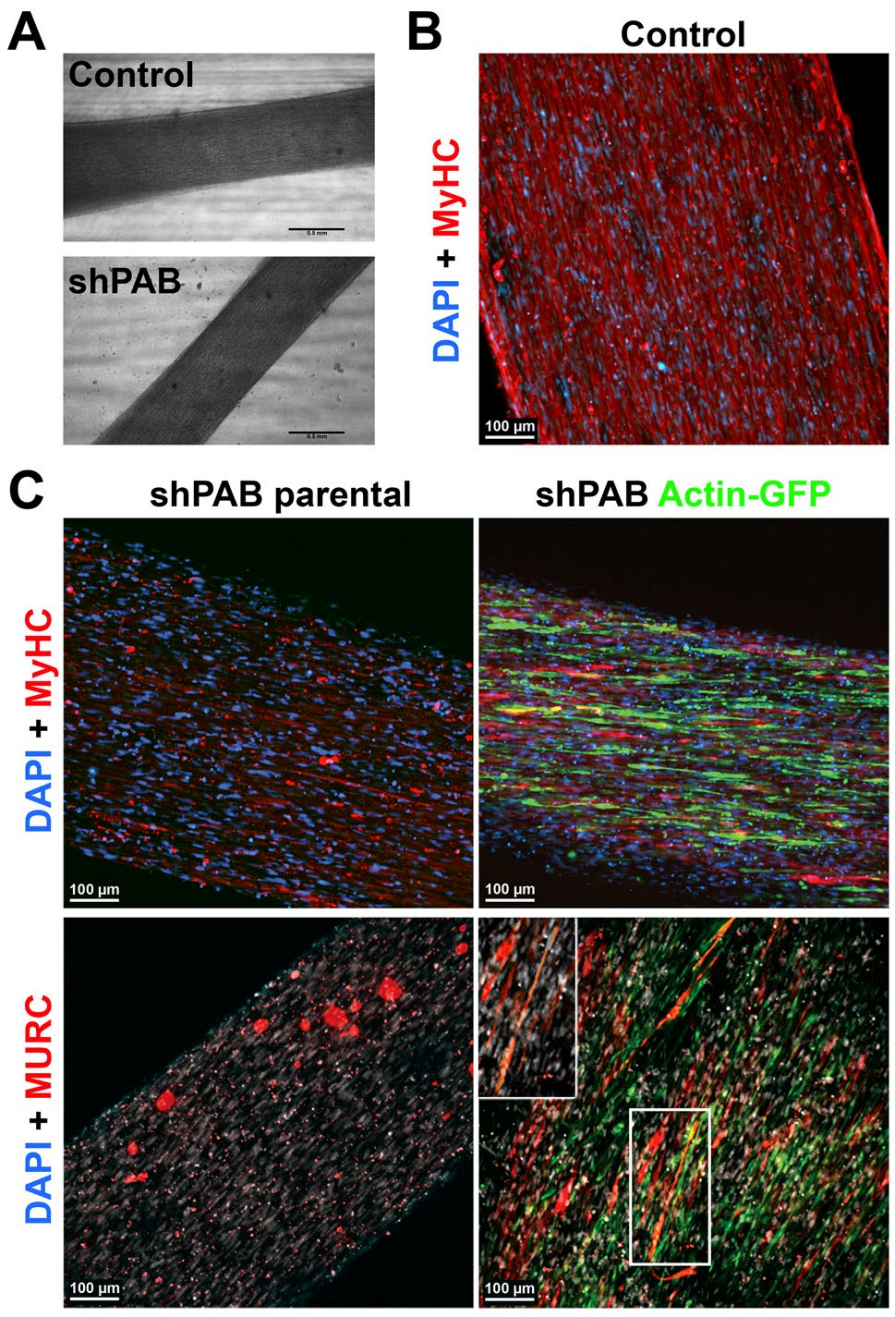
shPAB cultures form muscle bundles that lack myosin heavy chain expression. **A** Bright field images of muscle bundles generated from control and shPAB muscle cells. Scale bar is 500 µm. **B** MyHC staining in muscle bundle from control cells. **C** Maximum projection of muscle bundle staining using shPAB parental and shPAB + actin-GFP cells. MyHC or MURC are in red, GFP is in green. Nuclei are counterstained with Hoechst. Scale bar is 100 µm. Insert shows a magnification of MURC staining in multinucleated cells.

To validate and expand on the 2D cell culture results, we then employed the 3D muscle model, which has a better maturation of fused cells. We first assessed if muscle bundle could be generated from the shPAB muscle cells and then whether actin overexpression would be beneficial. After seven days of differentiation, muscle bundles were formed from both parental and shPAB cell cultures (Fig. 6A). Consistent with the 2D cell culture, the muscle bundles from control culture expressed MyHC (Fig. 6B). In the shPAB muscle bundle the expression of MyHC was clearly reduced compared to control (Fig. 6B and 6C). As the same fusion medium was used in both 2D and 3D cultures, but a synthetic matrix was added to the 3D muscle bundle, it suggests that cell fusion in the shPAB muscle bundle fusion is triggered by the synthetic matrix. Nevertheless, those muscle bundles may not form a contractible unit, as they express low levels of the MyHC. Noticeably, investigation of shPAB + actin-GPF muscle bundles showed an increase in MyHC expression (Fig. 6C). We then compared MURC expression in shPAB parental cells to shPAB + actin-GFP, and consistent with the 2D cell model, found that in the shPAB + actin-GFP muscle bundle MURC expression was clearly increased compared with shPAB parental (Fig. 6C). MURC staining in shPAB + actin-GFP was found in elongated cells (Fig. 6C). Together, the data suggests that actin overexpression is beneficial for expression of sarcomeric genes in shPAB cultures and partly restores the MURC expression.

## Discussion

Reduced PABPN1 levels cause muscle atrophy^6^. Atrophic muscles show alterations in cytoskeletal organization^41^. We found significant changes in expression levels of cytoskeletal proteins in shPab muscles. We show that in cell cultures with stable PABPN1 down-regulation the cytoskeletal structures, microtubules and actin filaments, lack orientation and organization. The small and round morphology of shPAB myoblasts implies an inability to obtain an elongated and polarized muscle cell shape. Both altered expression levels of cytoskeletal proteins and a disorganized architecture of the cytoskeletal filaments would influence the cell mechanical properties. Consistently, we found that both cell stiffness and cell surface tension are reduced in shPAB myoblasts. The defects in both cellular and mechanical features could be the reason for a failure in initiating cell fusion, as correct cytoskeletal organization is essential^42^.

Disrupted microtubule structures have been observed in muscular dystrophies such as Duchenne muscular dystrophy (DMD)^43^. In a recent study, disrupted microtubule organization was found to be associated with higher levels of tubulin proteins^44^. We also found elevated levels of tubulin proteins in the shPab proteome, including β-tubulin6 (Tubb6)^5^. Tubb6 knockdown was found to be beneficial for microtubule organization in the DMD mouse model^44^. Moreover, Tubb6 levels were higher in myofibers with central nuclei, suggesting a role in regeneration^44^. in shPab muscles PAX7 expression in myonuclei was increased compared with control^5^. An increase in PAX7 expression and in splitting myofibers suggests an increase in regeneration due to reduced PABPN1 levels. Here we show that cytoskeletal disorganization in shPAB 2D cell culture is linked to reduced myogenesis. Microtubule disorganization negatively affects cell fusion^20^. Targeting of microtubule stability, using small molecules drugs, was not beneficial for cell fusion^20,39^. Similarly, treatments affecting microtubule stability were not beneficial for cell fusion in shPAB cell cultures.

Consistent with a recent study showing that muscle aging in Caenorhabditis elegans is accompanied by a decline in the actin-cytoskeleton^17^, we also found reduced levels of actin in the shPab proteome. Stabilization of actin filaments is regulated by actin binding proteins^27,45^. Among those, CSRP3, PFN1, CFL1, ARP2/3 were found to be differentially expressed in shPab muscles^5^. Stabilization of actin filaments in shPAB cell cultures, using actin overexpression, led to an increased fusion index in shPAB muscle bundles. Consistently, actin was shown to be expressed at the fusion site and was suggested to be essential for fusion^46^. Nevertheless, actin overexpression did not completely restore myogenesis, as most shPAB MyHC expressing cells were smaller than in control fused cell cultures. This suggests that additional factors contribute to muscle atrophy in shPAB muscles.

Expression of the actin-binding protein CSRP3 (also known as Muscle LIM protein (MLP)) was suggested to maintain the actin-cytoskeleton^47,48^. A role for CSRP3 in myogenesis was also demonstrated^28,49^. Here we show that elevated CSRP3 levels were correlated with PABPN1 fold change in shPab muscles. Most prominent, CSRP3 expression highly correlated with MyHC-2A. MyHC-2A positive myofibers are more abundant in muscle atrophy and in shPab muscles^6^. This suggests that CSRP3 could mark atrophic myofibers. Increased CSRP3 levels were also found in different muscular dystrophies with muscle atrophy^28^. The role of CSRP3 in atrophic myofibers is yet to be studied.

The expression of another structural protein, MURC/Cavin-4, was also highly correlated with PABPN1 fold change in shPab muscles. MURC is a member of the caveola gene family that regulates plasma membrane invagination and consequently regulates plasma membrane-associated biological processes such as signaling, trafficking and mechanosensing^31^. The expression of both MURC and caveolin-3 (CAV3) is restricted to muscles^50^. MURC and Cav3 interact and form a complex at caveolae^31^. Same as MURC, Cav3 protein was also found to have higher expression in the shPab proteome. Both proteins are involved in myogenesis^30,31^. Loss-of-function CAV3 mutants may lead to different types of myopathies^51,52^. Mutations in MURC were found in patients with dilated cardiomyopathy^53^. MURC function is not fully understood, but it is suggested to stabilize CAV3 and to regulate calcium trafficking across the membrane^33,54,55^. We show that MURC is expressed with central nuclei and splitting myofibers, suggesting a role in regeneration. Consistently, increased MURC expression levels were also found in injury-induced muscle regeneration^30^. MURC is essential for muscle cell fusion^30^. Here we show that MURC distribution at the myotube membrane is regulated by the cytoskeleton. Destabilization of microtubules disrupts MURC localization at the myotube membranes, whereas stabilization of the cytoskeleton in shPAB myotubes, using actin overexpression, restores MURC’s myotube membrane localization. Moreover, MURC expression was partially restored at the myofibers’ boundaries in the shPAB-actin-GFP muscle bundles, which was absent in the shPAB muscle bundles. This further suggests that MURC may play a role in myopathies.

To summarize, we show a disordered cytoskeletal architecture in shPAB myoblasts and suggest a causative relation to reduced myogenesis. Furthermore, we found MURC as a novel marker for muscle regeneration. Stabilization of actin filaments, via actin overexpression, was beneficial for cell fusion in 2D or 3D models. The muscle bundle system seems beneficial for shPAB compared with the 2D cell culture system. The benefits of the muscle bundle model to study muscle atrophy should be investigated in future studies. The muscle bundle system seems to be beneficial to study PABPN1 regulation in muscle biology, as muscle bundles were formed from shPAB cells but the expression of sarcomeric and cytoskeletal genes was improper.

## Supplementary information

Supplementary Information

Supplementary Table S1

## Abbreviations

PABPN1: Polyadenylation binding protein nucleus 1
OPMD: Oculopharyngeal muscular dystrophy
Named shPab in mouse and shPAB in human: Reduced PABPN1 levels
APA: Alternative polyadenylation site
MyHC: Myosin heavy chain
DMD: Duchenne muscular dystrophy
