# Author Correction: Cytoskeletal disorganization underlies PABPN1-mediated myogenic disability

**DOI:** 10.1038/s41598-021-85459-0

**Published:** 2021-03-15

**Authors:** Cyriel Sebastiaan Olie, Erik van der Wal, Domagoj Cikes, Loes Maton, Jessica C. de Greef, I.-Hsuan Lin, Yi-Fan Chen, Elsayad Kareem, Josef M. Penninger, Benedikt M. Kessler, Vered Raz

**Affiliations:** 1 Human Genetics Department, Leiden University Medical Center, Leiden, The Netherlands; 2 IMBA-Institute of Molecular Biotechnology of the Austrian Academy of Sciences, Vienna, Austria; 3 VYM Genome Research Center, National Yang-Ming University, Taipei, Taiwan; 4 College of Medical Science and Technology, Taipei Medical University, Taipei, Taiwan; 5 Advanced Microscopy Facility, Vienna Biocenter Core Facilities, Vienna Biocenter (VBC), Vienna, Austria; 6 Target Discovery Institute, Nuffield, Department of Medicine, University of Oxford, Oxford, UK

Correction to: *Scientific Reports* 10.1038/s41598-020-74676-8, published online 19 October 2020

The original version of this Article contained errors in the spelling of the author Domagoj Cikes which was incorrectly given as Cikes Domagoj.

The Author Contribution section now reads:

C.S.O. and V.R. designed research; C.S.O. and L.M. performed cell culture experiments; D.C., E.K. and J.M.P. performed Biomechanical experiments; E.W., J.C.G. and V.R. generated muscle cell bundle; C.S.O. and B.M.K. generated the shPab proteome; I.L. and Y.C. generated the aging transcriptome; C.S.O. and V.R. wrote the paper; All authors commented on the manuscript.

These errors have now been corrected in the PDF and HTML versions of the Article and the Supplementary Information file that accompanies the Article.

