# Supplementary material for: Cytoskeletal disorganization underlies PABPN1-mediated myogenic disability
Source: Sci Rep. 2020 Oct 19;10:17621. doi: 10.1038/s41598-020-74676-8 (PMC7572364; doi:10.1038/s41598-020-74676-8)
Supplement: Supplementary file 1 — Supplementary Information [file 41598_2020_74676_MOESM1_ESM.docx]

**Supplementary information**

**Cytoskeletal disorganization underlies PABPN1-mediated myogenic disability**

Cyriel Sebastiaan Olie^1^, Erik van der Wal^1^, Domagoj Cikes^2^, Loes Maton^1^, Jessica C. de Greef^1^, I-Hsuan Lin^3^, Yi-Fan Chen^4^, Elsayad Kareem^5^, Josef M. Penninger^2^, Benedikt M. Kessler^6^ and Vered Raz^1*^

^1^Human Genetics department, Leiden University Medical Center, The Netherlands.

^2^IMBA-Institute of Molecular Biotechnology of the Austrian Academy of Sciences, Vienna, Austria.

^3^VYM Genome Research Center, National Yang-Ming University, Taiwan.

^4^College of Medical Science and Technology, Taipei Medical University, Taiwan.

^5^Advanced Microscopy Facility, Vienna Biocenter Core Facilities, Vienna Biocenter (VBC), Vienna, Austria.

^6^Target Discovery Institute, Nuffield Department of Medicine, University of Oxford.

***Supplementary Tables***

**Table S1. Cytoskeletal protein differentially expressed in shPab muscles.**

**A.** Indicates the PABPN1 fold change across the four mice that was detected by western blot. **B**. Shows the significantly affected cytoskeletal proteins in shPab muscles as defined in our previous paper (Olie et al., *iScience* **12**, 318, 2019). Data of one protein was not available (NA) because it was not detected. Proteins highlighted in green present the protein with a fold change of 1.5 or higher and 0.67 or lower. The correlation was calculated over the fold changes detected by the mass spectrometer and the PABPN1 western blot as presented in S1A and Fig. S1. The go term ontology (BP = biological process, CC = cellular component or MF = molecular function) indicated by DAVID bioinformatic resources (https://david.ncifcrf.gov/) was used to determine a role in actin-binding or actin cytoskeletal organization (highlighted in gold) or a role in microtubules (light blue). Correlation for each protein was calculated over the PABPN1 fold change (S1A) and the respective protein fold change in this table. **C.** Table shows the cytoskeletal genes that were common between S1B and the transcriptome of aged mouse skeletal muscles (Lin et al., *BMC Genetics* **19**, 55, 2018). Highlighted in green (Csrp3 and Murc) have a fold change higher than 1.5 in both studies. Genes highlighted in gray were present in both studies but had a fold change lower than 1.5.

***Supplementary Figures***

**
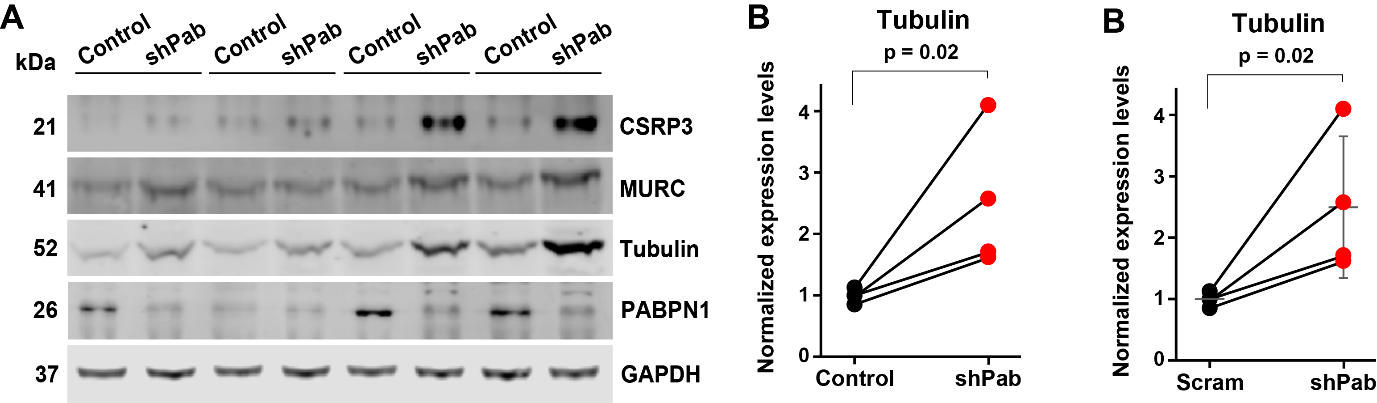
**

**Fig S1. CSRP3, MURC and tubulin expression in paired tibialis anterior (control) versus shPab) muscles**

**A.** Western blots show CSRP3, MURC and tubulin expression in control and shPab muscles. GAPDH was used as loading control. **B.** Western blot quantification. Paired dot-plot shows tubulin expression levels after normalization to loading control, N=4 mice. Statistical significance is assessed with a ratio-paired t-test.


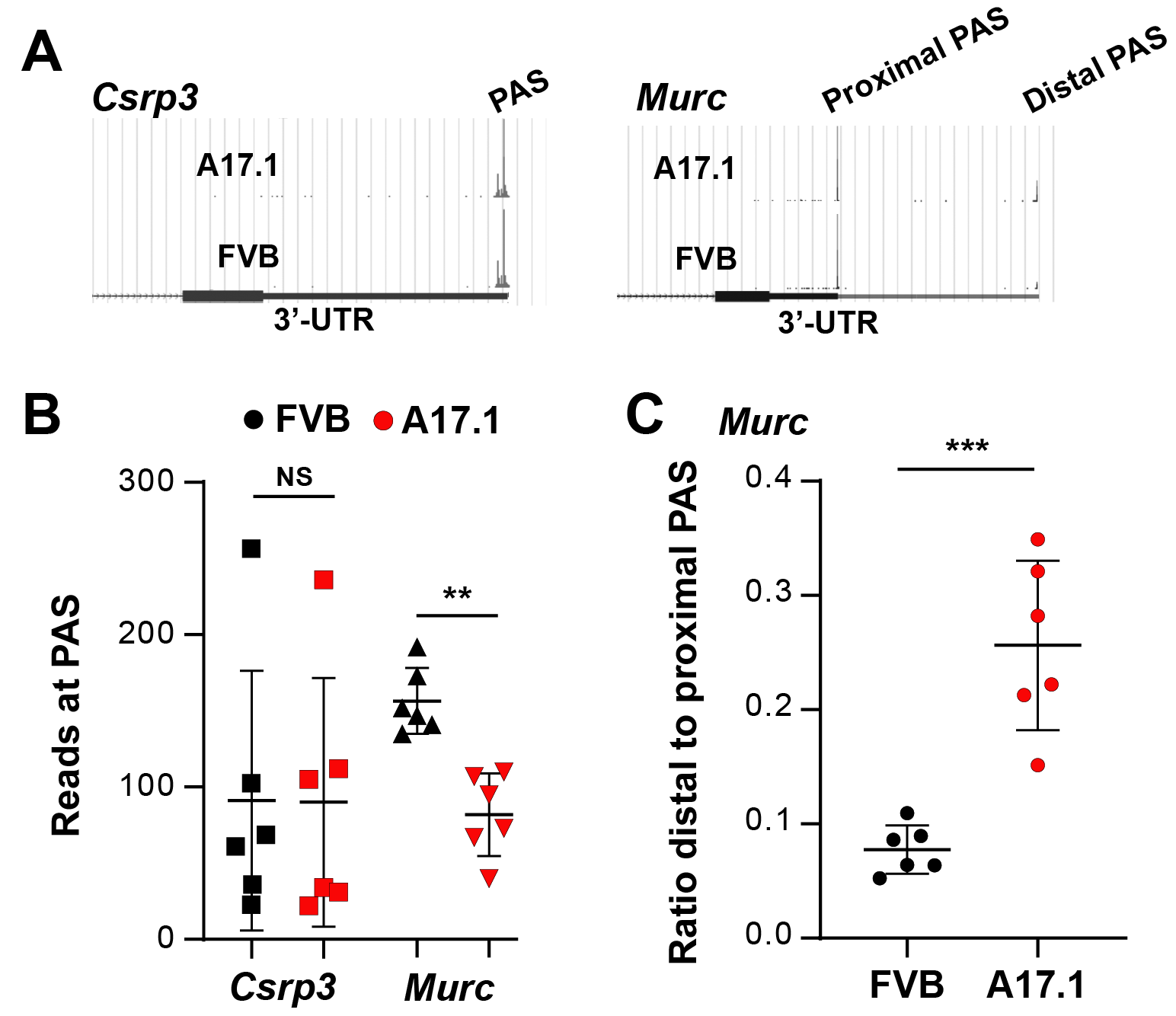


**Fig S2. Alternative polyadenylation utilization in *Csrp3* and *Murc* transcripts.** Data was taken from our study (de Klerk et al., 2012), which was carried out in tibialis anterior muscles comparing reads at the polyadenylation site in FVB (black), control, versus A17.1 (red), a mouse model over expressing the expanded PABPN1. **A.** A UCSC screenshot of *Csrp3* and *Murc* 3’-UTR showing reads at polyadenylation sites (PAS) in FVVB and A17.1 mouse. **B.** Dot plot shows the number of reads each mouse (N=6). In *Csrp3* only one polyadenylation site was found. In *Murc* the reads shown are from the proximal polyadenylation site. **C.** Dot plot shows the ratio of reads between distal to proximal polyadenylation site in *Murc*. Statistical analyses were carried out on six mice. The significance of a student’s t-test is depicted as follows: p<0.01 **; p<0.005 ***; not significant (NS).


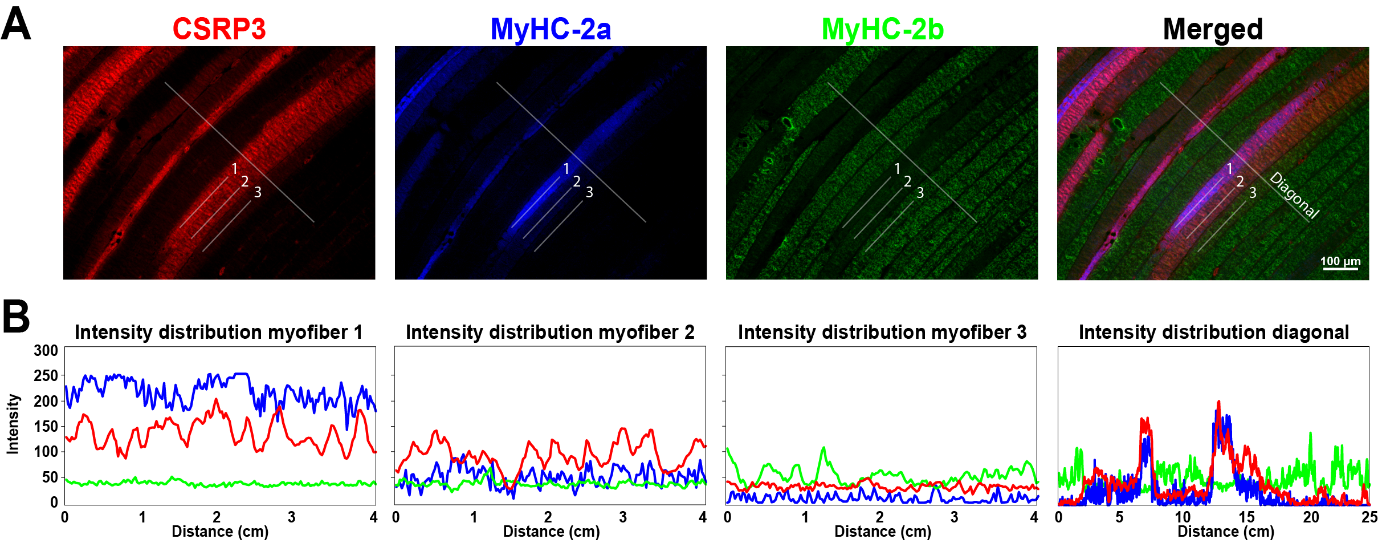


**Fig S3. CSRP3 co-localization with MyHC-isoforms**

**A.** Longitudinal section showing MURC and MyHC-2A or 2B spatial distribution in TA muscle from control. Transparent lines indicate myofibers used for distribution plots. Scale bar is 100 μm. **B.** Distribution plots of three individual fibers and over multiple fibers (diagonal). Based on eye evaluation, myofiber #1 expresses both MyHC-2A and CSRP3, myofiber #2 expresses CSRP3, and myofiber #3 MyHC-2B.


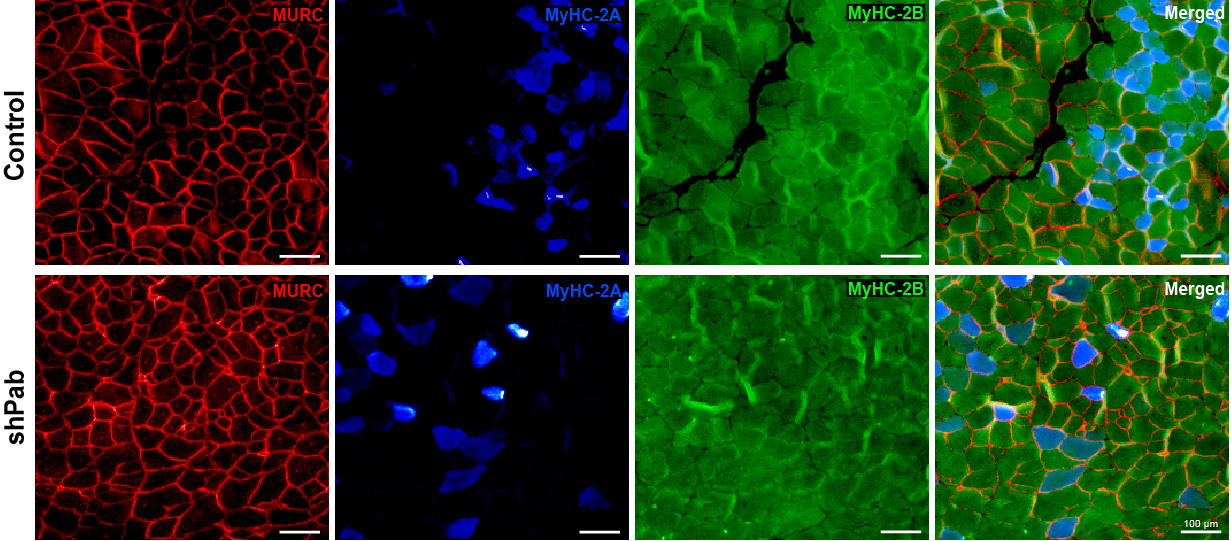
**Fig S4. MURC co-localization with MyHC-isoforms**

Representative images of MURC and MyHC-2A or 2B in control or shPab TA muscle cross-sections. Scale bar is 100 μm.


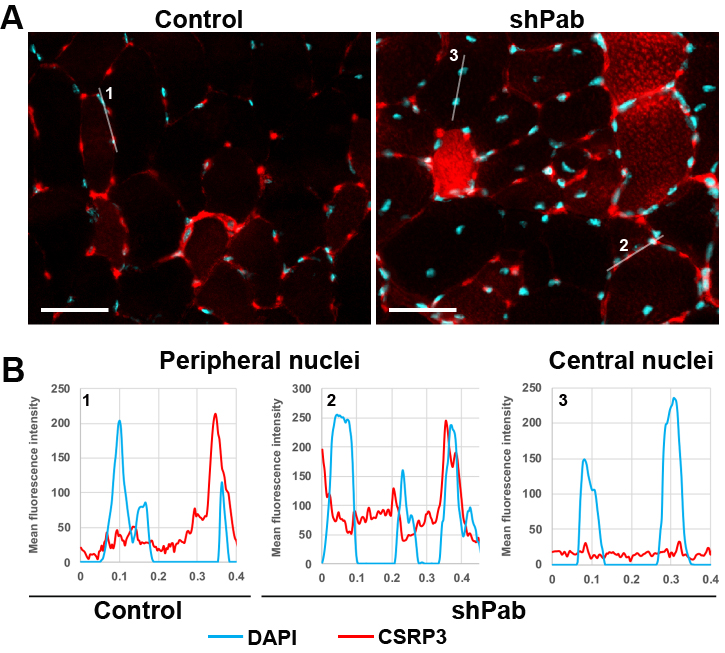


**Fig S5. CSRP3 co-localization with myonuclei**

**A.** Images of CSRP3 and DAPI in control or shPab TA muscle cross-sections. Scale bar is 50 μm. **B.** Intensity distribution plots show spatial overlap between CSRP3 and DAPI along the white lines, marked as 1 to 3. Two lines are from peripheral regions (#1 and #2), and #3 shows central nuclei. CSRP3 shows some overlap with peripheral myonuclei but not with central nuclei.


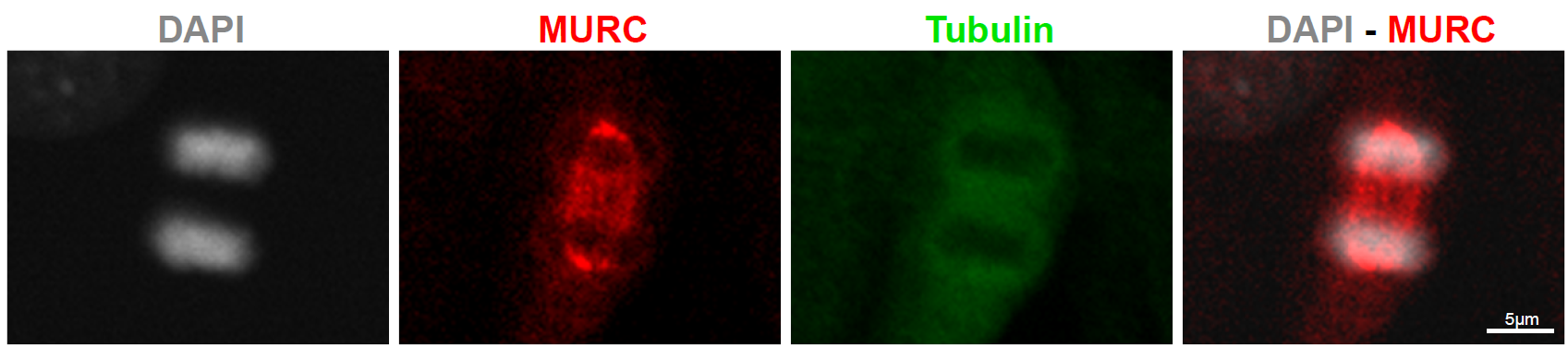


**Fig S6. MURC localization in a dividing myoblast.**

Images of DAPI (grey), MURC (red) and tubulin (green) of a dividing control myoblast. Scale bar is 5 μm.

**
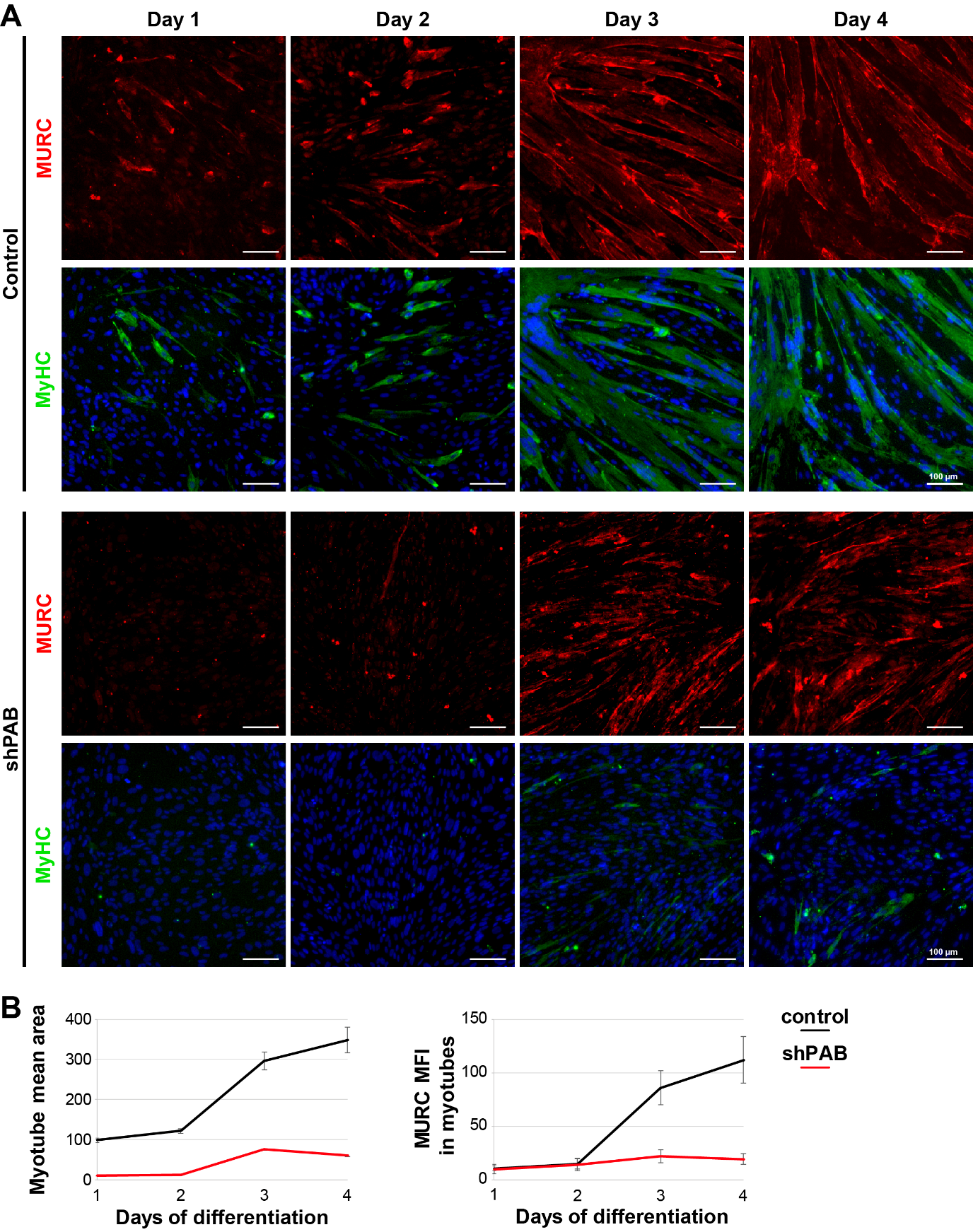
**

**Fig S7. MURC expression during myogenic differentiation.**

**A**. Representative fluorescence images show MURC expression after one, two, three or after four days in differentiation medium. Upper panel shows images for control cells and lower panel for the shPAB cell cultures. Scale bar is 100 µm. **B**. Graphs show the MyHC area (myotube) and the MURC mean fluorescence intensity within the MyHC positive objects (called myotubes) during the four days of differentiation. The number of cells were equal between control and shPAB cultures and did not change throughout the four days.


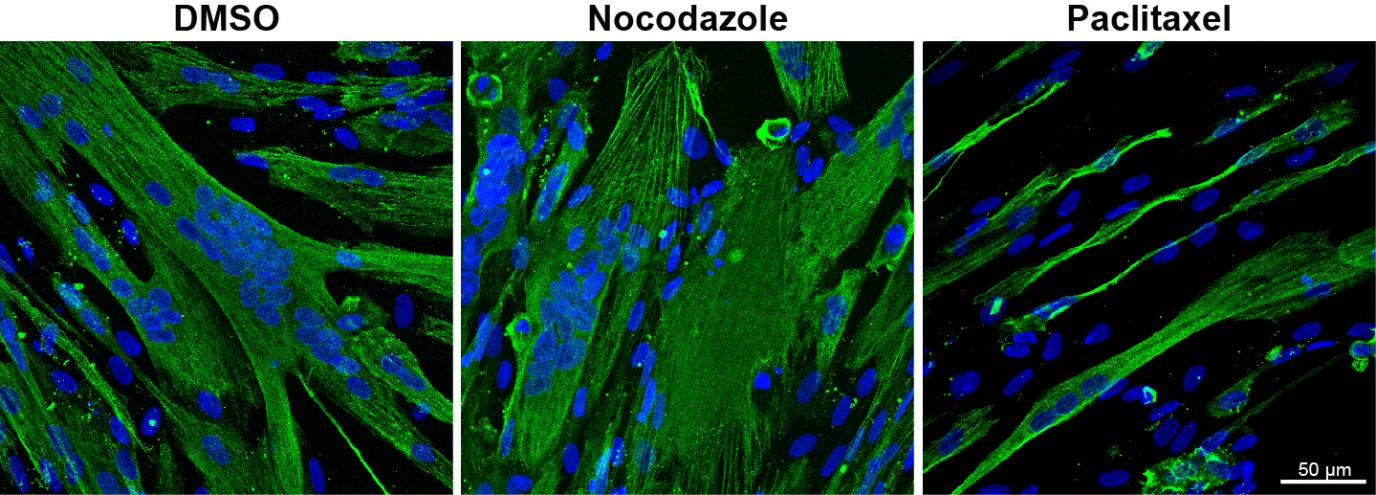


**Fig S8. Microtubule treatment on fully matured myotubes.**

Control myoblasts were fully differentiated for four days. The fully matured myotubes were subsequently treated with nocodazole or paclitaxel for two hours. Myosin heavy chain is stained in green and nuclei in blue. Scale bare is 50 µm.


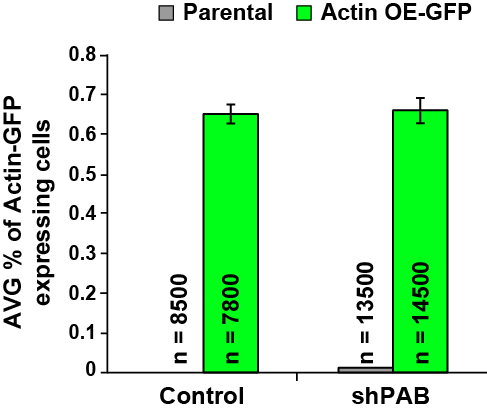


**Fig S9. Percentage of GFP-positive cells after actin-GFP transduction of control or shPAB cell cultures.**

Parental cells were used as negative control for imaging in the GFP channel. N shows the average number of cells that were counted. Average and standard deviation are from three replicates.


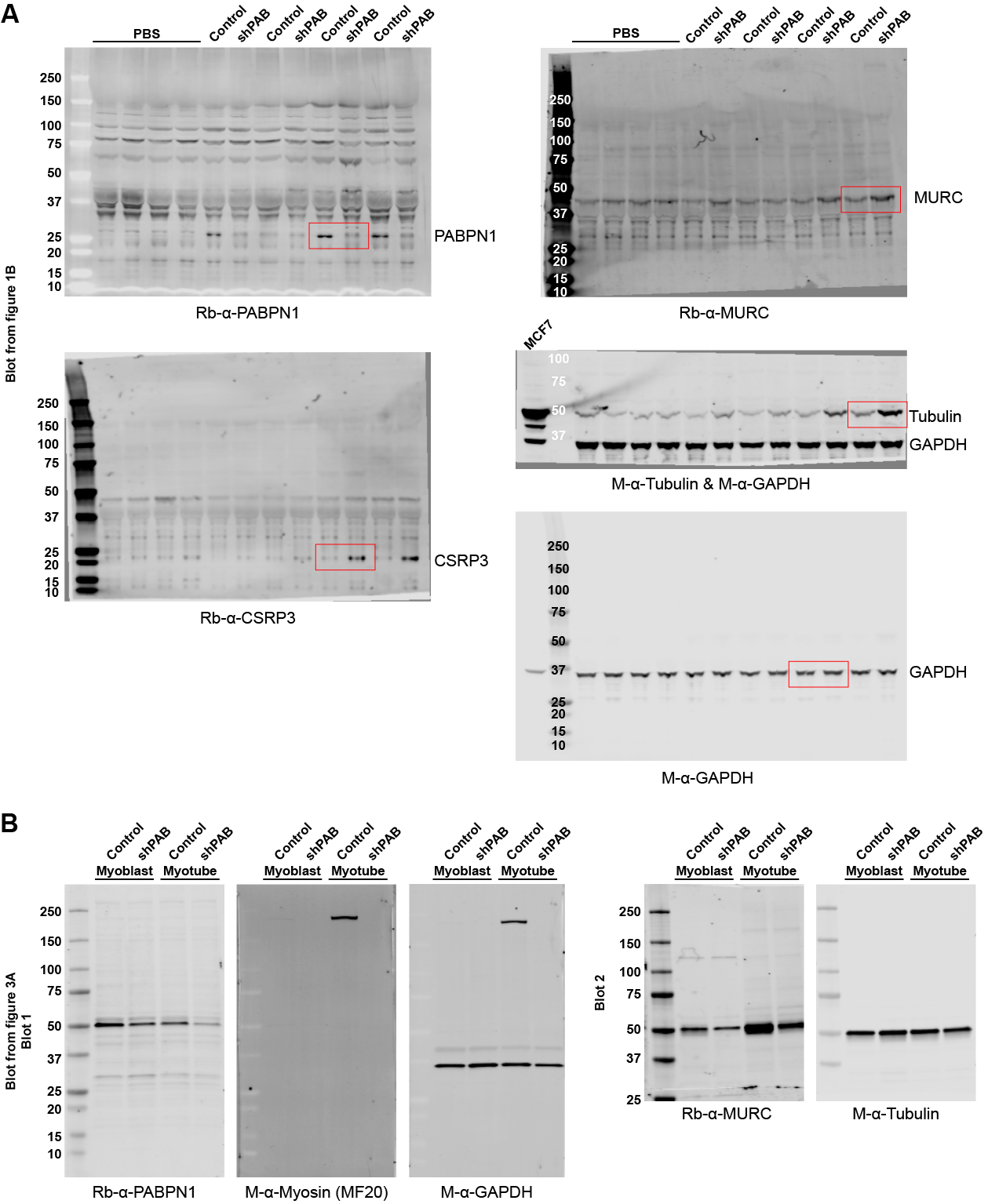


**
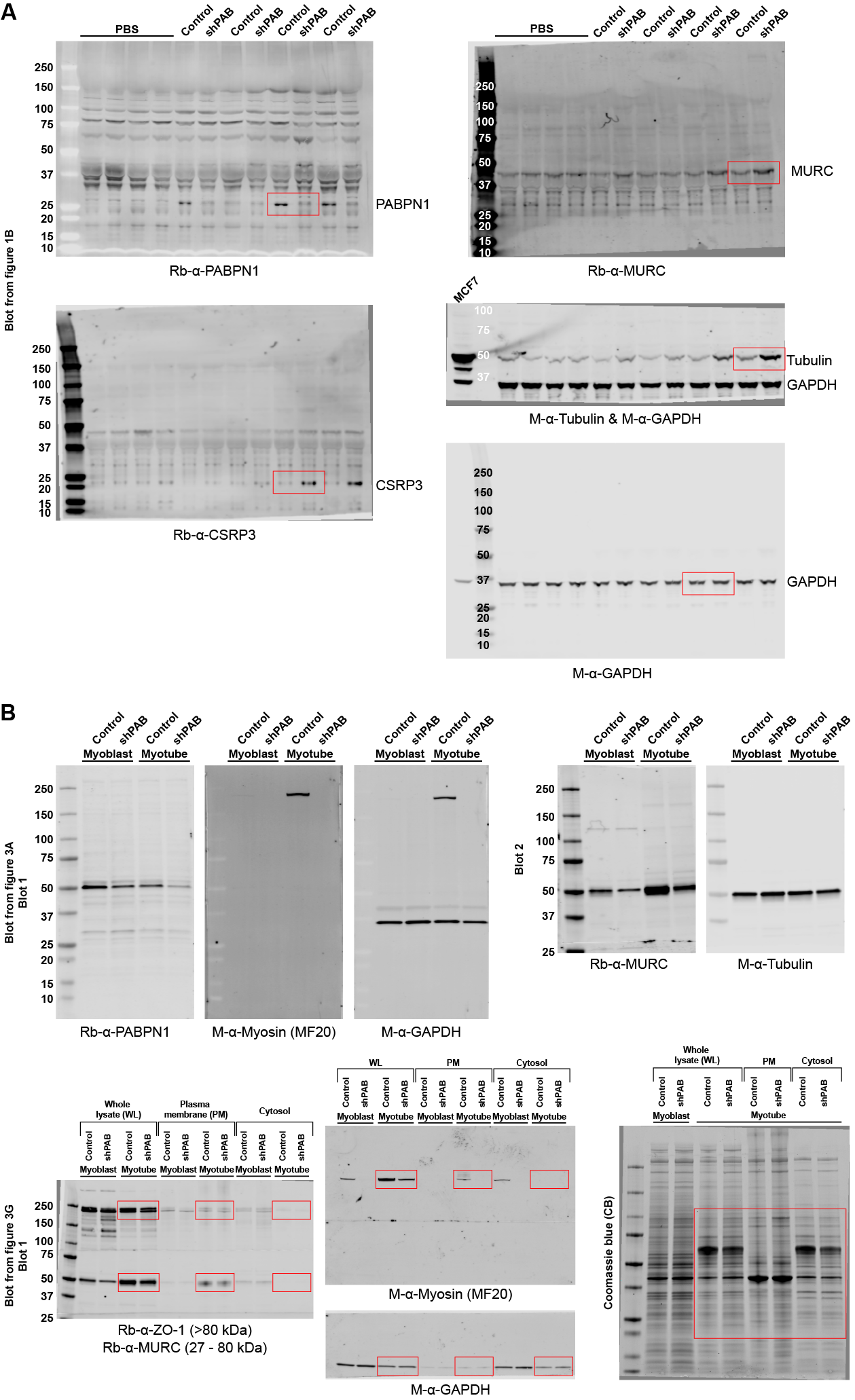
**

**Fig S10. Full length western blots. A.** Western blot of the PBS, scrambled shRNA and PABPN1 shRNA injected mouse muscles. Red boxes indicate the cropped blots which are shown in figure 1B. **B.** Western blots of the 7304.1 human muscle cell cultures. Blot 1 has been blotted for PABPN1 and imaged in the 700nm channel, subsequently this blot was then blotted for Myosin in the 800nm channel and then for GAPDH as a loading control. Blot 2 has first been blotted for MURC and secondly for Tubulin, in the 700nm and 800nm channel, respectively. The subcellular fractionation blot was first incubated for ZO-1 and MURC and subsequently for GAPDH and Myosin. The regions presented in the main text figures are highlighted with a red rectangle.
